# Metabolomics Analysis and Diagnosis of Lung Cancer: Insights from Diverse Sample Types

**DOI:** 10.7150/ijms.85704

**Published:** 2024-01-01

**Authors:** Simin Liang, Xiujun Cao, Yingshuang Wang, Ping Leng, Xiaoxia Wen, Guojing Xie, Huaichao Luo, Rong Yu

**Affiliations:** 1 College of Medical Technology, Chengdu University of Traditional Chinese Medicine, Chengdu, 611137, China.; 2 State Key Laboratory of Southwestern Chinese Medicine Resources, School of Pharmacy, Chengdu University of Traditional Chinese Medicine, Chengdu, 611137, China.; 3 Department of Clinical Laboratory, Sichuan Cancer Hospital & Institute, Sichuan Cancer Center, University of Electronic Science and Technology of China (UESTC), Chengdu, China.

**Keywords:** Lung cancer, Lung Neoplasms, Biomarkers, Biological Marker, Metabolomics, Metabonomics, Chromatography, Mass spectrometry

## Abstract

Lung cancer is a highly fatal disease that poses a significant global health burden. The absence of characteristic clinical symptoms frequently results in the diagnosis of most patients at advanced stages of lung cancer. Although low-dose computed tomography (LDCT) screening has become increasingly prevalent in clinical practice, its high rate of false positives continues to present a significant challenge. In addition to LDCT screening, tumor biomarker detection represents a critical approach for early diagnosis of lung cancer; unfortunately, no tumor marker with optimal sensitivity and specificity is currently available.

Metabolomics has recently emerged as a promising field for developing novel tumor biomarkers. In this paper, we introduce metabolic pathways, instrument platforms, and a wide variety of sample types for lung cancer metabolomics. Specifically, we explore the strengths, limitations, and distinguishing features of various sample types employed in lung cancer metabolomics research. Additionally, we present the latest advances in lung cancer metabolomics research that utilize diverse sample types. We summarize and enumerate research studies that have investigated lung cancer metabolomics using different metabolomic sample types. Finally, we provide a perspective on the future of metabolomics research in lung cancer. Our discussion of the potential of metabolomics in developing new tumor biomarkers may inspire further study and innovation in this dynamic field

## 1. Introduction

Lung cancer is the cancer with the highest mortality rate worldwide. According to recent statistics, in 2022, an average of nearly 1,700 people died every day in the United States, with more than 350 deaths attributed to lung cancer each day, which is more than the combined number of deaths from breast cancer, prostate cancer, and pancreatic cancer 1.

Lung cancer is characterized by the uncontrolled proliferation of lung tissue cells. Uncontrolled proliferation that is not treated on time can quickly spread to tissues near the lungs and eventually to other parts of the body. Lung cancer is divided into central and peripheral types according to their location. According to its biological characteristics, lung cancer is divided into non-small-cell lung cancer (NSCLC) and small-cell lung cancer (SCLC), of which NSCLC accounts for about 80%. NSCLC includes large-cell carcinoma, adenocarcinoma, squamous cell carcinoma, and other types 2, 3. The means of the diagnosis of lung cancer include laboratory tumor markers, imaging examination and histopathologic analysis of biopsy 4.

Lung cancer has high mortality rate as it is difficult to make an accurate and effective early diagnosis. The majority of patients are diagnosed with advanced-stage lung cancer. Statistics show that about 15% of patients are diagnosed during the initial stages (stages I-II). Effective treatment is possible at this stage, with a 5-year survival rate of about 60%. In contrast, almost 60% of patients are diagnosed with metastatic disease (stage IV), prognosis is very poor, with a 5-year survival rate below 5% 5. Other studies have found that 2-year progression-free survival is a reliable surrogate for overall survival in lung cancer, with a 24-month progression-free survival of 40.3% and overall survival of 69.4% after treatment with chemotherapy plus surgery 6. In recent years, low-dose computed tomography (LDCT) has been widely used in the early screening of lung cancer. Compared with chest radiography, LDCT screening can reduce the risk of lung cancer mortality by approximately 20% 7-9. However, LDCT screening can lead to high false-positive results. A previous meta-analysis found that across all trials and cohorts, 20% of individuals in each screening round screen positive and require some level of follow-up. In the final results, only approximately 1% of individuals had lung cancer 10. A high false-positive rate will not only waste the resources of diagnosis and treatment but also cause unnecessary panic to people. Therefore, there is an urgent need to find sensitive and specific markers for the diagnosis of lung cancer to supplement the deficiency of imaging examinations.

Metabolomics is a major branch of system biology. It is an emerging discipline and technology in the era of “post genomics.” It is one of the most active fields of life science research worldwide. Since the introduction of the concepts of metabolomics, it has aroused great interest from scientists worldwide. Scientists have realized that changes in the genome are not necessarily reflected in the biological phenotype; that is, they do not affect the life system. The production of a series of small-molecule metabolites is the final result of various reactions and changes in organisms and the human body. They can directly and accurately reflect the physiological and pathological states of organisms. If biology is regarded as information science, the study of metabolomics provides information on the biological information flow, between the levels of genes and cells, and plays a connecting role in the transmission of biological information. Therefore, some scientists have concluded that genomics tells you what may happen, proteomics tells you what will happen, and metabolomics tells you what has happened 11, 12.

Compared with other omics methods, metabolomics also has the following characteristics. First, small changes at the genetic level are amplified at the metabolite level, making it easier to detect physiological and pathological changes in vivo. In addition, metabolomics technology requires a relatively complete database of metabolite information, but since the number of metabolites in vivo is much smaller than the number of whole genome sequencing data, the construction and improvement of metabolomics database is relatively easy 13, 14. Because metabolomics reflects the terminal effect under the comprehensive action of various factors and is highly integrated, metabolomics has great potential for identifying reliable biomarkers of various diseases 15.

The aim of this article is to provide an academic account of the application of metabolomics in lung cancer research, with a specific focus on its advancements in identifying new potential metabolic biomarkers for lung cancer diagnosis. To achieve this, we begin by briefly introducing and discussing the metabolic pathways impacted by lung cancer. Subsequently, we describe the commonly used instrumental platforms in lung cancer metabolomics and explore the various sample types and sources employed in such research.

The main points of this review center on the advantages, disadvantages, and characteristics of various sample types utilized in lung cancer metabolomics research. We carefully selected and analyzed a large number of representative articles in recent years to summarize and discuss the latest research results of lung cancer metabolomics in different sample types. Finally, we look forward to the future prospects of metabolomics research in lung cancer. By providing a comprehensive overview of the state-of-the-art advances in lung cancer metabolomics research, we hope to inspire further research and development in this vital field.

## 2. Main metabolic pathways of differential metabolites

As a malignant tumor, lung cancer disrupts multiple metabolic substances and pathways in the human body. Firstly, the rapid proliferation of lung cancer cells inevitably leads to an increase in anaerobic oxidation, resulting in responsive changes in a series of metabolites associated with glycolysis. Secondly, due to the extensive growth of lung cancer cells, there is a significant increase in the consumption of lipids, especially phospholipids, which serve as important building blocks for cell membranes. Furthermore, many amino acids and their derivatives are also involved in the altered metabolic pathways caused by lung cancer. For example, glutamine serves as an important nutrient source for cancer cells, while serine acts as a precursor for purine nucleotides and plays a crucial role in DNA synthesis and cell proliferation. The following is a classification introduction of the metabolic substances and pathways affected by lung cancer 16, 17.

### 2.1 Glucose metabolism

As early as the middle of the last century, Otto Warburg, a famous physiologist in Germany, discovered that unlike normal cells, which mainly rely on mitochondrial oxidative phosphorylation for energy supply, most cancer cells mainly rely on glycolysis for energy supply. This phenomenon was later called the “Warburg effect,” which has been discussed and verified in many generations 18. Studies have shown that due to the increased energy expenditure of cancer tissues, the concentration levels of a series of enzymes related to glucose metabolism such as phosphofructokinase, 3-phosphoglycolaldehyde dehydrogenase, phosphoglycolate kinase-1, pyruvate kinase, and pyruvate dehydrogenase increase significantly 19.

Because cancer cells use the glycolytic pathway for energy supply, the accumulation of lactate affects the lactate cycle 20. Lactic acid is the most commonly detected metabolite, whether using blood samples or tissue samples from lung cancer patients 21-25. In recent years, a study has used carbon isotopes to label glucose and glutamine and to analyze metabolic pathways using isotopic tracers. The study confirms that glucose in cancer patients is indeed substantially converted to lactate and that glucose and glutamine are the major sources of carbon in the TCA cycle 26. In addition, Some research teams pointed out the relationship between tumor aggressiveness and the level of lactic acid elevation; that is, the more aggressive the tumor, the more dependent the tumor metabolism is on the glycolysis pathway, and thus the higher the level of lactic acid detected 27, 28.

### 2.2 Lipid metabolism

Lipid metabolism changes in tumor patients are manifested as endogenous lipid hydrolysis and fatty acid oxidation enhancement, increased triglyceride conversion, decreased exogenous triglyceride hydrolysis, and increased plasma free fatty acid concentration. Increased lipolysis and oxidation of fatty acids leads to decreased body fat storage and weight loss. Therefore, fat consumption is one of the main features of tumor cachexia 20. Previous studies have reported that the levels of unsaturated lipids, LDL, and VLDL in the serum of cancer patients are significantly reduced, which may be due to the proliferation and invasion of tumors, thus causing a large amount of lipid consumption 22, 23.

Choline is a metabolite commonly found in the body. Compared with healthy individuals, the concentration of choline in the serum of lung cancer patients is reduced as it is the precursor of phospholipids in the cell membrane, which is consumed through the rapid division and reproduction of malignant tumors 23. Phosphatidylcholine is composed of choline and phosphatidylic acid, and is the main component of the cell membrane. The concentration of phosphatidylcholine in serum gradually increases with invasion and metastasis of cancer cells 24, 25.

Sphingomyelin is the main component of the cell membrane. Its metabolites, such as sphingosine, sphingosine-1-phosphate, and ceramide, are bioactive signaling molecules that can be used as first or second messengers to regulate cell growth, differentiation, aging, and apoptosis, and many other important signal transduction processes 29. To explore the role of plasma S1P and ceramide levels in the diagnosis of lung cancer, Alberg et al. performed a nested case control study. Increased plasma levels of S1P and total ceramide increase lung cancer risk. Furthermore, we suggest that disorders of sphingolipid metabolism and production of S1P may be linked to lung cancer pathogenesis and could be potential biomarkers for early diagnosis of lung cancer 30.

Lysophosphatidylethanolamine (LPE) is a lysophosphatidylcholine involved in cell signal transduction in vivo. Yu et al. found that LPE can be used as a diagnostic marker for NSCLC. Compared with those of healthy controls, LPE concentrations were elevated in the plasma of patients with NSCLC, especially in patients with adenocarcinoma. In addition, the concentrations of lecithin ethanolamine were also significantly elevated in the plasma of patients with lung cancer 31.

### 2.3 Amino acid metabolism

Due to the increase in glycolysis, alanine concentrations in lung cancer patients will naturally increase. The uptake of leucine, valine, and isoleucine is also increased in lung tumors as they are required for the production of TCA cycle intermediates 32, 33. Phenylalanine is a precursor of tyrosine and neurotransmitters such as dopamine, norepinephrine, and adrenaline. The serum phenylalanine level in lung cancer patients usually significantly increases due to the downregulation of genes involved in phenylalanine metabolism in tumor cells 34.

Glutamine is an important component of the amino acid metabolism. Glutamine is a substance that cancer cells depend on for their survival and reproduction. It does not only play an important role in the synthesis of various proteins but also participates in the synthesis of fats and nucleotides. Glutamine is an important nitrogen and carbon source for a variety of biochemical pathways in the body. When the body lacks local glucose supply, glutamine can also be used as the raw material for cancer cells to obtain energy. Glutamine and water are broken down into glutamic acid and ammonia, respectively, under the action of glutaminase. Glutamate and pyruvate then form α-ketoglutarate and alanine, respectively, under the action of pyruvate transaminase. A-ketoglutarate then enters the TCA cycle to produce energy. Glutamine hydrolysis produces ammonia and glutamate to balance the pH of tumor cells, which may explain the high glutamate levels observed in the NSCLC patient group 22, 23, 35. Further, glutamine can also be used to determine the prognosis of cancer. When NMR technology was used to analyze patients' serum metabolites, patients with higher glutamine levels survived longer, while those with lower glutamine levels survived shorter 35.

Cysteine and glutamic acid are important GSH components. Fahrmann et al. found that the concentrations of cysteine and glutamate were significantly increased in patients with lung adenocarcinoma by comparing tumor tissues with control tissues. In addition, many enzymes associated with glutathione biosynthesis, glutathione cycling, allobiotic metabolism, and nitrogen balance are significantly increased 36. Serum histidine and threonine levels are reduced in lung cancer patients due to the increased utilization of the glycine, serine, threonine, and pyrimidine pathways 23, 37. Some researchers have pointed out that serine is a key node in cancer cell metabolism. The serine synthesis pathway provides serine for protein synthesis in cancer cells. The serine synthesis pathway, together with glycolysis and the 1C metabolic pathway, forms a crucial metabolic network for tumorigenesis. Specifically, the serine synthesis pathway is an important destination for the glycolysis intermediate 3-phosphoglycerate. Therefore, monitoring serine metabolism is of great significance for studying the occurrence and development of lung cancer as well as improving the treatment of lung cancer 16.

### 2.4 Summary of metabolic markers

In recent years, an increasing number of lung cancer metabolomics experiments have not only provided new potential markers for the early diagnosis of lung cancer, but also studied the metabolic pathways affected by lung cancer to gain a deeper understanding of its pathogenesis 38, 39. It should be noted that,even with the same type of lung cancer, patients with different disease stages will show different metabolic characteristics. Zhang et al. indicated that the serum glutamine concentration in lung cancer patients was significantly higher than that in normal controls 22. By contrast, Puchedes-Carrasco et al. observed a reduction in serum glutamine levels in lung cancer patients compared with that in normal controls 23. When the similarities and differences between the two studies were compared, the stages of lung cancer cases used in the study differed. The former study focused on patients with early-stage lung cancer, while the latter study included patients with all stages of lung cancer, and the proportion of patients with advanced-stage lung cancer was relatively large. Therefore, the serum glutamine concentration is related to the stage of lung cancer, and glutamine tends to increase in the early stage of lung cancer due to the stress response. In the advanced stage of lung cancer, patients often present with cachexia, a wasting syndrome, and glutamine is largely decomposed as a functional substance; therefore, the blood shows a downward trend. Therefore, we must pay attention to the different stages of the same disease may produce some obvious differences.

In general, the common metabolic alterations in lung cancer metabolomics studies include glycolysis pathway 40, tricarboxylic acid cycle pathway 24, pentose phosphate pathway 41, etc. Unfortunately, these metabolic alterations are often reported in other tumors and thus lack specificity. Some less mentioned pathway alterations may merit continued attention and in-depth study, such as biodegradation pathways of aromatic compounds 42, phospholipid metabolism 43, putrescine and spermidine generation and transformation 44. Exploration and in-depth discussion of the alterations in these metabolic pathways may lead to a deeper understanding of the mechanism of the disease.

## 3. Platform of lung cancer metabolomics

### 3.1 Nuclear magnetic resonance (NMR)

NMR is the main technique used in metabolomics research and has become one of the schools of metabolomics research. The NMR detection and analysis platform is characterized by nondestructive and unbiased sample detection without complicated sample preprocessing. It has good objectivity, easy quantification, simple analytical conditions, and good reproducibility. However, compared with MS, it has relatively low sensitivity and limited dynamic range; hence, it is difficult to simultaneously determine the metabolites with large concentration differences coexisting in biological systems. To improve the sensitivity of NMR techniques, the field intensity was increased using cryogenic probes and microprobes 45, 46. In recent years, high-resolution magic angle spinning proton magnetic resonance spectroscopy detection has been increasingly used, which can directly detect in vitro tissues, without physical or chemical processing of samples, to obtain higher-resolution spectrograms. Especially when the tissue structure is not damaged, high-resolution spectral information can be obtained to screen the metabolites, which can move freely in the cell solute with appropriate rotation speed 47, 48.

### 3.2 Mass spectrometry (MS)

MS analysis is characterized by its high sensitivity and is one of the most important and widely used platforms for metabolomics analysis 49, 50. In addition, MS can detect ions without protons or carbon, such as metal ions. As a detection technology, MS can overcome certain limitations of NMR. MS is usually combined with chromatography and is characterized by the separation of complex mixtures into single components. With the development of MS and its combination technology, the rapid analysis and identification of multiple compounds have been achieved. Therefore, an increasing number of researchers have applied MS to metabolomic studies 51-54. In recent studies, isotopic tracer technology using an MS platform has been developed to explore the metabolism and signal pathways more intuitively and accurately, thereby obtaining new and valuable biological insights 55.

#### 3.2.1 Gas chromatography-mass spectrometry (GC-MS)

GC-MS is suitable for the separation and analysis of samples that are stable and easy to gasify, particularly for the separation of homologues and isomers 56, 57. The sensitivity, efficient separation, high-resolution, and good repeatability of GC-MS make it suitable for the analysis of complex metabolic mixtures. More importantly, this method has a reference and comparative standard spectrum library among different laboratories worldwide, which can be used for the characterization of metabolites. GC-MS has been criticized for the need for derivatization of many samples, which increases the time required for sample preparation. For metabolites with low volatility, GC-MS is either unable to measure or requires complex sample pretreatment steps. These shortcomings restrict the application of this detection method to a certain extent.

#### 3.2.2 Liquid chromatography-mass spectrometry (LC-MS)

Compared with GC - MS, LC - MS has higher sensitivity and wider testing scope, so the LC - MS in metabonomics analysis in recent years has been widely used. LC-MS avoids the complex sample pretreatment in GC-MS. Because front-loaded liquid chromatography allows easy separation of mixtures, LC-MS can be used to detect potential markers in complex biological samples 58, 59.

Conventional C18 columns are commonly used for the separation of components, but for hydrophilic metabolites, they are poorly retained in reversed-phase chromatography. In order to solve this problem, Hydrophilic Interaction Chromatography was used for the analysis and detection of hydrophilic substances. Different types of ionization methods can be selected according to the metabolites to be measured. Atmospheric pressure chemical ionization method is the earlier electrical method developed, while matrix-assisted laser desorption-ionization method is increasingly used in current metabolomics research due to its superior effect 60-62.

#### 3.2.3 Capillary electrophoresis-mass spectrometry (CE-MS)

CE-MS is another characteristic and noteworthy technology in metabolomics research. Compared with GC-MS, which is only suitable for volatile substances, CE-MS can easily analyze non-volatile substances without cumbersome derivatization steps. Compared with LC-MS, CE-MS is particularly suitable for the separation of polar and charged compounds because, in principle, the technology is based on the charge-to-mass ratio of the compounds. Therefore, CE-MS is frequently utilized in metabolomic research and can be complementary to the above methods. CE-MS has many advantages. It does not require complicated pretreatment schemes, it consumes very few organic solvents, it does not require organic solvents, it uses cheap fused silica capillaries instead of expensive chromatographic columns, and the analysis and separation of components is convenient, cheap, and fast. However, CE has low stability and sensitivity, and the supporting schemes for combined use with MS are also less than those of GC-MS and LC-MS 63, 64.

### 3.3 Summary of instrument platforms

NMR and MS, the two pillar platforms in metabolomics research, are widely used in the study of physiological and pathological metabolism. NMR does not require cumbersome sample preparation, and its detection is convenient and fast. It does not destroy the sample, and the same sample can be tested repeatedly without sample loss. NMR is the only metabolomics platform that can be used for in vivo detection. One of the greatest criticisms of NMR is its low sensitivity (relative to mass spectrometry), and some metabolites at low concentrations may be difficult to detect. MS has been increasingly used in metabolomics research in recent years. MS is often combined with chromatographic techniques to enable better separation of metabolites. GC-MS technology has high sensitivity and a good separation effect, and is very suitable for the detection of volatile and less polar substances. The GC-MS technology is relatively stable and has good repeatability. In terms of substance identification, relatively mature and complete public databases are available for reference and comparison. However, GC-MS can only detect non-volatile and polar substances. Although the volatility of the tested substances can be enhanced by derivatized pretreatment, the detection of such substances remains a technical problem. To a large extent, LC-MS technology can compensate for the deficiencies of GC-MS. It can detect difficult-to-detect volatile and polar substances, and can flexibly replace chromatographic columns and columns with different characteristics according to the experimental needs. During the mobile phase, the range of detectable substances is significantly increased. The main limitation of LC-MS technology is that the compatibility of public databases is not strong enough, and material characterization is more dependent on the established local databases or standard substances, which is cumbersome to operate and has high cost ratio. CE-MS is rarely used; this method is especially suitable for the separation of charged compounds and macromolecular substances and can be used as an effective supplement to the above methods. If energy and funds are sufficient, the multi-platform combined analysis can cover more metabolite species, which is conducive to the screening of metabolic markers for lung cancer diagnosis and identification (Table 2).

## 4. Sample types of lung cancer metabolomics

Various types of metabolomic samples are available. Generally speaking, the common sample types in metabolomics experiments for the diagnosis of lung cancer are as follows: blood (including serum and plasma), urine, saliva, expiration (organic volatiles), alveolar lavage fluid, skin, tissues, and cells. Each type of specimen has its own unique pretreatment method, and its functions also differ.

### 4.1 Blood

Blood is one of the most common and popular sample types in metabolomics research. The blood metabolome undergoes dynamic changes at various stages of the organism's own genetic replication, transcription, and translation, and is continuously regulated by the body, reflecting the individual's health or disease status. These changes are induced by endogenous or exogenous metabolites, such as those provided by diet or oral medications. Therefore, blood metabolomics has been widely applied in the exploration of potential disease biomarkers, pharmacodynamics, and the discovery of drug targets, providing relevant clues for the diagnosis and treatment of diseases 65, 66.

#### 4.1.1 Serum

Serum samples, as a widely utilized sample type in metabolomics research, hold significant importance. The preparation procedure for serum samples is relatively straightforward, involving the natural coagulation of blood followed by centrifugation. Such simplicity allows for the application of diverse analytical methods, including mass spectrometry, chromatography, and nuclear magnetic resonance, thus enabling comprehensive metabolomic analysis. Serum samples serve as valuable resources in capturing various metabolic alterations associated with diseases. Utilizing these samples, researchers are able to explore and identify potential disease markers. The rich metabolomic information obtained from serum not only aids in deciphering the underlying metabolic changes induced by diseases but also offers promising avenues for disease diagnosis 67, 68.

Published in 2018, Hu J et al. collected serum samples from 43 healthy individuals and 39 patients with advanced-stage NSCLC, tested the samples using NMR, and analyzed the differences in metabolites shown on hydrogen NMR spectra. The serum levels of glutamate, glycoprotein, lactic acid, phenylalanine, alanine, tyrosine, proline, and tryptophan were Significantly elevated, while the levels of glutamine, taurine, glucose and glycine were reduced in patients with NSCLC compared with those in healthy individuals. Therefore, serum metabolites are potential biomarkers for NSCLC diagnosis 42.

In a scholarly publication from 2020, Chen et al. conducted a study involving 142 patients with NSCLC and 159 healthy controls. Serum samples were collected from both groups and subjected to analysis using the LC-MS platform. After comparison and statistical analysis, the results showed that there were 35 significantly different metabolic biomarkers between the two groups. Compared with the healthy population, the serum levels of metabolites such as C16-Sphinganine, Phytosphingosine, Sphinganine, Capric acid, and Arachidate were increased in lung cancer patients, while metabolites such as lysoPC(18:2), acyl-carnitine C10:1, and Inosine were decreased. The researchers performed a combined analysis of the ROC curves of the differential metabolites and ultimately identified a separation model containing six differential metabolites: lysoPC(18:2), acyl-carnitine C10:1, L-Tryptophan, Indoleacrylic acid, Inosine, and Hypoxanthine. The sensitivity of this model was 95.7%, and the specificity was 95.0%. In addition, the research team further accessed relevant transcriptomic data through the TCGA database. The metabolomic and transcriptomic data can be matched and validated with each other, revealing disturbances in metabolic pathways such as glycolysis, amino acid metabolism, phospholipid metabolism, and fatty acid metabolism in NSCLC patients 69.

The following year, Schult et al. collected serum samples from 79 NSCLC patients and 79 healthy controls, and used high-resolution magic angle spinning (HRMAS) proton magnetic resonance spectroscopy (MRS) to measure differences in metabolites between the two groups. The experiment showed that changes in organic acids, amino acids, carnitine, sugar phosphates, vitamins, coenzymes, nucleosides, nucleobases, and derivatives could establish a model for early diagnosis of lung cancer. The metabolic pathway disorders affected by lung cancer were most evident in glycolysis and tricarboxylic acid cycle metabolism. Furthermore, this model could also predict the 5-year survival rate of lung cancer patients 70.

In a scholarly publication by Jiaoyuan et al. in 2023, the researchers aimed to investigate the serum samples of 193 patients diagnosed with NSCLC and 243 healthy controls. Notably, 70% of the samples from each group were randomly selected for analysis and modeling, and the remaining 30% were used as a validation set to test the modeling results. In this study, the serum was detected using a mass spectrometry analysis platform, and a total of 278 statistically different metabolites were found by comparing and analyzing the metabolite concentrations between the two groups. Among them, the serum levels of metabolites significantly increased in lung cancer patients included 11,12-Epoxy-(5Z,8Z,11Z)-icosatrienoic acid, Cholic acid, 11-Deoxyprostaglandin, Glycocholic acid, and Docosahexaenoic acid ethyl ester, which were more than 2 times higher in lung cancer patients than in healthy controls. In contrast, metabolites such as 2,4-Dihydroxybenzoic acid, Salicylic acid, and Piperine were significantly decreased in the serum of lung cancer patients. Algorithms such as Random Forest (RF) and Support Vector Machine (SVM) were used to establish a model for lung cancer diagnosis, which was tested using samples from the validation set. The sensitivity of the random forest model was 74%, and the specificity was 92%, indicating that the model had high specificity and could be used for exclusive diagnosis. The sensitivity of the support vector machine model was 83%, and the specificity was 89%, indicating that the overall diagnostic accuracy was high. Pathway analysis found that the most significant disturbances in lung cancer patients occurred in the phenylalanine, linoleic acid, and bile acid metabolism pathways 71.

#### 4.1.2 Plasma

Plasma is a vital sample type that plays a crucial role in metabolomics research. It constitutes the supernatant fraction obtained from blood that has been mixed with an anticoagulant, retaining fibrinogen. While the majority of the components present in plasma are similar to those found in serum, including hormones, inorganic ions, amino acids, sugars, lipids, and various other metabolites, the response intensity of certain substances in nuclear magnetic resonance or mass spectrometry may differ from that observed in serum. Despite these differences, plasma remains widely deployed as a key sample type in metabolomics analysis. By comparing metabolic differences between plasma derived from patients versus healthy individuals, valuable insights for disease diagnosis and treatment can be gleaned. The information gleaned from plasma samples may help identify potential disease markers and facilitate the development of precision medicine approaches 65, 72.

In a scholarly publication from 2018, Peng et al. conducted a study aimed at predicting the response of patients with lung cancer to chemotherapy based on platinum. The researchers collected plasma samples from 43 patients with varying chemotherapeutic outcomes and analyzed them using LC-MS-based metabolomics. The study employed a rigorous methodology involving multivariate statistical, pathway, and correlation analyses, leading to the identification of eight biomarkers that demonstrated significant correlations with the efficacy of platinum-based chemotherapy 73.

According to a recent scholarly publication in 2021, Zheng et al. aimed to identify potential serum diagnostic biomarkers for lung cancer. The researchers conducted an analysis of plasma samples obtained from both lung cancer patients and healthy individuals using gas chromatography-mass spectrometry (GC-MS). Multiple algorithms were applied to screen and identify candidate biomarkers. The study findings revealed the diagnostic value of several differential metabolites, such as oleic acid, 2-hydroxybutyric acid, cholesterol, and inositol, in accurately diagnosing lung cancer 38. In the same year, Sarlinova M et al. designed an interesting metabolomics experiment using plasma samples. Plasma samples were collected from 132 patients with primary lung cancer and 47 patients with secondary lung cancer. Metabonomics analysis showed that there was no significant difference in plasma metabolites between primary lung cancer and secondary lung cancer.Compared with the healthy control group, the common characteristics of the lung cancer group were significantly increased glucose, citrate, acetate, 3-hydroxybutyrate, and creatinine levels, and decreased pyruvate, lactic acid, alanine, tyrosine, and tryptophan levels. The differential metabolites mentioned above can be used as auxiliary modes for diagnosing lung cancer 74.

In the exploration of disease biomarkers, most researchers prefer untargeted metabolomics as it is a comprehensive and unbiased detection strategy that facilitates the discovery of novel metabolic markers. However, if the target metabolites to be studied and detected have been pre-defined, targeted metabolomics is a better choice. Untargeted metabolomics can only achieve relative quantitation based on the response intensity of signal peaks, whereas targeted metabolomics enables precise quantitative analysis by setting standards and isotopes. Cao et al. collected plasma samples from 128 lung cancer patients to explore biomarkers that can distinguish between squamous cell lung cancer and adenocarcinoma of the lung. They used an LC-MS analysis platform to conduct targeted metabolomics detection based on endogenous metabolites. The results showed that 2-(methylthio)ethanol, cortisol, D-glyceric acid, and N-acetylhistamine could effectively distinguish between squamous cell lung cancer and adenocarcinoma of the lung. The diagnostic model established using these four biomarkers had a sensitivity of 92.0% and a specificity of 92.9%. In addition, through KEGG database analysis of metabolic pathways, it was found that the differences between the two types of lung cancer mainly occurred in the riboflavin metabolism pathway and the steroid hormone biosynthesis pathway 75.

#### 4.1.3 Dried blood spots (DBS)

DBS testing, in which whole-blood samples are collected on paper, has certain advantages over conventional methods of blood sample collection. Dried blood plaques have been used to measure phenylalanine levels since the 1960s 76, 77. From the perspective of sample collection, it requires less amount of blood and is less invasive compared whole-blood collection. As regards sample preservation, dried blood plaque is more stable than traditional blood samples; thus, its storage and transportation costs are much lower than that of whole blood 78. Owing to the simplicity and high stability of the DBS method, it has been widely applied in disease diagnosis 79, 80. In recent years, scholars have proposed that using 80% acetonitrile as the extraction solvent and injecting internal standards after the chromatographic column is beneficial for metabolomics research based on DBS. Tests have shown that using this method can effectively improve the quality of detected data 81.

In a study published in 2020, Yu et al. collected DBS for metabolomic studies of lung cancer. The DBS samples from 37 SCLCs, 40 NSCLCs, and 37 controls were analyzed. A combination of five differential metabolites was selected to establish a lung cancer diagnosis model, and the diagnostic accuracy of the combination was 95% for male SCLC patients and 94% for female SCLC patients by receiver operating characteristic curve analysis. In subsequent studies, a validation cohort comprising 78 individuals was used to further evaluate the performance of the discriminant model. Results showed that the accuracy rates of the developed discriminant model were 91% for men and 81% for women 82.

#### 4.1.4 Discussion of blood sample studies

Which sample type of serum and plasma is more suitable for the study of metabolomics has aroused a wide discussion among researchers 83, 84. When using serum or plasma for metabolomics studies, the results produced by these two sample types will inevitably differ to some extent due to the differences in the preparation methods and production principles of the two samples 85. The serum does not require the addition of anticoagulants, which avoids the matrix effect caused by anticoagulants and the potential interference of anticoagulants on metabolic activities 86. However, the formation of blood clots requires a long wait time. During this process, some unstable metabolites may be degraded, resulting in the loss of potential marker metabolites 87. Another influencing factor is that platelets release a variety of compounds into the serum during coagulation, including peptides, hypoxanthine, and xanthine 88, 89. Therefore, a standardized process should be followed during serum sample collection, as well as during the coagulation of all samples. Based on the above reasons, using plasma for metabolomic analysis is a better strategy. The collected plasma no longer needs to undergo additional steps related to blood coagulation and can be directly centrifuged and stored after mixing, which makes the experimental results obtained by plasma analysis more repeatable 90.

The choice of anticoagulants must be considered when using plasma in metabolomic studies. The experimental results of some researchers showed that there was only a slight difference between the results of common anticoagulant heparin and ethylenediaminetetraacetic acid (EDTA) in the metabolomic analyses 87. However, some scholars believe that EDTA does not only inhibit blood coagulation but also suppress the production of magnesium ion-dependent enzymes in red blood cells, such as glycolytic enzyme hexokinase, thus making plasma more suitable for metabolomic applications 91. Anticoagulants are usually pre-added to vacuum-sampling tubes. Goldberg et al. compared the differences in metabolomic studies of lung cancer patients when using Streck and heparin tubes for blood collection; blood samples from 42 patients with suspected lung cancer were collected in Streck and heparin tubes, and were used for metabolomic analysis using LC-MS. Statistical analysis showed that leucine and other 18 kinds of compound concentration in Streck tube is higher, and 15 compounds such as LysoPC concentration is higher in the heparin tube 92.

Some researchers believe that serum is the preferred sample type for metabolomics studies, because plasma contains anticoagulants, and common anticoagulants such as EDTA, citrate, etc., can interfere with the metabolic fingerprint generated by NMR spectroscopy 65. A recent study showed that among the metabolites associated with the diagnosis of the disease, including acylcarnitines, bilirubin, nucleosides, etc., the concentrations of these metabolites in serum were significantly different from their detected concentrations in plasma samples supplemented with different anticoagulants^93^ For this reason, when conducting multi-center, large-sample metabolomics studies, it is important to consider the interference and impact caused by different types of blood samples collected by different institutions.

### 4.2 Urine

Urine is an important type of sample in metabolomic studies. Using urine as a metabolomic sample has a series of advantages. First, the collection of urine is noninvasive; hence, it is easier to obtain, and patients or volunteers are more willing to cooperate 94. Moreover, changes in urine samples include the joint effects of endogenous and external environmental factors, such as diet, exercise, and metabolism of the intestinal microbiota. Therefore, urine samples can be used for a comprehensive process. However, when using urine as a sample, we must also pay attention to the following problems. First, urine samples must be collected at a fixed time according to the needs of the test (e.g., collecting morning urine). Moreover, it may contain cells (such as red blood cells, white blood cells, and epithelial cells), bacteria, tubular cells, fat droplets, mucus filaments, and other components 95. Hence, it is necessary to add an appropriate amount of preservative to the urine sample to ensure its stability. In addition, because urine samples are usually collected by the subjects themselves, the researchers should inform the subjects in advance of the precautions for collecting urine. Finally, it should be noted that similar to the two studies mentioned below, the urine biomarkers of lung cancer found by metabolomics are still in the laboratory research stage, and their real application in clinical diagnosis or prognosis evaluation of lung cancer may need several years of exploration and practice 66.

In a study published in 2014, Mathé et al. collected urine samples from 1005 subjects, including 469 patients with non-small cell lung cancer and 536 healthy controls. The samples were analyzed using an LC-MS instrument platform, and the results were statistically analyzed. It was found that the levels of creatinine riboside and N-acetylneuraminic acid (NANA) were significantly elevated in cancer patients compared to healthy controls. The researchers then collected another 80 urine samples from patients with non-small cell lung cancer and 78 from healthy controls to verify the reliability of the two biomarkers. The validation results were consistent with the initial experiment. The authors further pointed out that the higher the levels of creatinine riboside and N-acetylneuraminic acid, the poorer the prognosis of lung cancer patients 96.

In a study published by Seow et al. in 2019, a case-control research design was implemented. They selected 275 female lung cancer patients from a pool of non-smokers and chose 278 healthy female controls of similar age for comparison. Urine samples were collected from both groups and analyzed using mass spectrometry and NMR techniques. The study results revealed a protective association between 5-methyl-2-furoic acid and lung cancer risk. This metabolite is partially derived from soy-related dietary products. The authors speculated that bioactive compounds present in soy may possess antioxidant and anti-inflammatory properties, which could inhibit tumor growth, invasion, and induce cell apoptosis.Furthermore, exposure to environmental pollutants, particularly long-term exposure to traffic-related air pollution, increases the risk of developing lung cancer. Disruptions in certain systemic metabolic pathways, such as one-carbon metabolism and oxidative stress response, have also been associated with lung cancer risk 97.

Zhao et al. analyzed the changes of metabolic substances in the urine of lung cancer patients and explored its potential pathogenesis using bioinformatics. Based on the case-control experiment, urine metabolites were detected by LC-MS platform. Statistical analyses were performed using Multivariate to identify potential lung cancer markers. Thirty-five potential markers were found. Five key markers were found to correlate well linearly with serum biochemical indices after screening and optimization. In addition, further studies have shown that glutamine metabolism disorder and amino acid imbalance are closely related to the occurrence and development of lung cancer 98.

In a study published in 2021, Ahmed et al. collected 29 pairs of urine samples and 32 pairs of serum samples from early-stage lung cancer patients before and after surgery. The samples were analyzed using mass spectrometry and NMR, and the results showed that the changes in urinary metabolites were greater than those in blood before and after surgery in lung cancer patients. In urine samples, the concentrations of Leucyl proline and isopentenyladenine significantly decreased after surgery, with Leucyl proline decreasing to 1/625 of its pre-surgery concentration and isopentenyladenine decreasing to 1/31 of its pre-surgery concentration. However, the concentration of N6-methyladenosine increased to 27 times its pre-surgery concentration after surgery. The researchers believed that surgery, as a direct intervention, divided the metabolic state of patients into having cancer (pre-surgery) and not having cancer (post-surgery), and the metabolic biomarkers identified in this study could be used as a basis for monitoring treatment effects and possibly for diagnosing the presence or absence of lung cancer 99.

### 4.3 Saliva

Saliva is secreted by salivary glands and has functions such as cleaning the oral cavity, moisturizing the oral cavity, and pre-digesting food. As a biological fluid that can reflect the physiological and pathological status of the human body, saliva has metabolic components similar to tissue fluid and is a sample type worthy of attention in clinical diagnosis. In addition, saliva sample collection is non-invasive and sampling is very convenient, with high patient compliance, making it suitable for early disease screening of large-scale populations 100, 101.

In a study published in 2021, Nijakowski et al.'s systematic review paper introduced the research progress of metabolomics studies of saliva samples in the diagnosis and treatment of multiple tumor diseases 102. Specifically, in the field of metabolomics in lung cancer, Jiang et al collected saliva specimens from 89 patients with early-stage LC, 11 patients with advanced-stage LC, and 50 healthy controls. To screen for potential saliva biomarkers for early diagnosis of lung cancer, researchers used an ultra-low noise TELDI-MS platform to detect the metabolic profiles of saliva samples. By using statistical methods to analyze metabolic differences among groups, researchers combined 23 altered saliva metabolites to establish a model that can distinguish between LC patients and healthy controls, with a sensitivity of 97.2% and specificity of 92% 103.

According to a paper published in 2022, Takamori et al. collected saliva samples from 41 patients with LC and 21 patients with benign lung lesions (BLL). To explore the differential metabolites between the two groups of samples, researchers used a capillary electrophoresis mass spectrometry platform to test and analyze saliva metabolic products. In addition, in the process of data analysis, a multiple logistic regression (MLR) model was established to evaluate the distinguishing ability of each saliva metabolite.The analysis detected ten significantly different metabolites between the two saliva sample groups from LC and BLL patients. The concentration of tryptophan (Trp) was significantly lower in the saliva samples from the LC group than the BLL group. The concentrations of choline, thymine, cytosine, phenylalanine (Phe), leucine (Leu), isoleucine (Ile), lysine (Lys), and tyrosine (Tyr) in the saliva samples from the LC group were higher than those from the BLL group, but the difference was not significant. The MLR model combination of diethanolamine, cytosine, lysine, and Tyr had a good distinguishing ability for distinguishing between BLL and LC (AUC = 0.729) 104.

### 4.4 Exhaled breath (volatile organic compounds)

Volatile organic compounds (VOCs) are a collective term for a class of organic matter present in the air at room temperature in the form of vapor, and the products that are excreted after trans-pulmonary metabolism are called endogenous VOCs, which are the products of human metabolism; thus, dynamic changes in its concentration, as well as in its composition, can reflect changes in the human body in terms of physiology, with relevance to cancer or other diseases 105. As early as 1971, Linus Pauling, a Nobel laureate, conducted a preliminary study of VOCs in respiration. The gas exhaled through the lungs is a complex mixture of approximately 250 VOCs 106. Breathing is also closely associated with lung function. It can collect metabolic information that reflects a variety of lung diseases, including lung cancer. Moreover, it is a fast, convenient, and completely noninvasive sample collection method, and patients or volunteers are more willing to cooperate.

Sakumura et al. used GC-MS to analyze the incorporation of lung cancer patients and healthy controls, and performed subsequent statistical analysis based on multiple combinations of lung cancer-associated VOCs to diagnose lung cancer. Using GC-MS analysis, 68 different VOCs were detected, including methanol, CH3CN, isoprene, and 1-propanol. The support vector machine algorithm was used to optimize and select a set of combinations containing five VOCs. This combination is sufficient to achieve a screening accuracy of 89.0%; therefore, it can be used to design and develop a breath sensor analysis system for lung cancer diagnosis 107.

### 4.5 Bronchoalveolar lavage fluid (BAL)

BAL is an important test for the diagnosis of respiratory and pulmonary diseases. Although BAL is a nearly noninvasive test, saline lavage often induces cough and other discomforts; therefore, 2% lidocaine is usually injected for tracheal local anesthesia. Bronchoalveolar lavage fluid is rich in metabolically related biochemical substances and has a unique value in the diagnosis of respiratory diseases, including lung cancer 108.

Leblic et al. collected bronchoalveolar lavage fluid from lung cancer patients and screened these samples for presence of different metabolites by GC-MS analysis combined with bioinformatics analysis. The results show that glycerol and phosphate can be used not only in the diagnosis of lung cancer, but also in the prognosis of the disease 43.

### 4.6 Skin

The skin is the largest organ of the human body and also the most superficial. As an unconventional specimen type in metabolomics research, sampling of skin surface specimens is often overlooked by researchers. Changes in the composition of substances such as sweat, sebum, and stratum corneum emitted by the skin can reflect alterations and abnormalities in metabolic pathways in the body, making the metabolic substances on the skin surface an external manifestation of the body's physiological or pathological state 109, 110. The generation of sweat is easily disturbed by internal or external factors. For example, factors such as the temperature of the environment in which the subject is located or the subject's own exercise status can have unstable effects on sample collection. Therefore, it is necessary to unify these conditions as much as possible when collecting sweat samples 111.

There are various ways to collect sweat, one of which is the use of hydrogel micro-patches, which are patches made of polytetrafluoroethylene with internal cavities to absorb sweat 112, 113. Overall, sweat collection is a non-invasive, easy-to-collect, and highly compliant sampling method. As an unconventional sample, sweat metabolomics research has great research space and prospects 109

Calderon et al. established a model based on sweat metabolites to distinguish between lung cancer patients and healthy individuals. The researchers collected sweat samples from 41 lung cancer patients and 55 healthy controls. Notably, among the healthy controls, 24 were active smokers and 31 were non-smokers. The liquid chromatography-mass spectrometry platform was used to detect the concentration of metabolites in the two groups of sweat samples. The research team used PanelomiX to develop two models. The first model aimed for high specificity and was composed of three metabolites: suberic acid, a tetrahexose, and a trihexose. Analysis showed that this model had a specificity of 80% and a sensitivity of 69%. The second model aimed for high sensitivity and was composed of three metabolites: nonanedioic acid, a trihexose, and the monoglyceride MG(22:2). Analysis showed that this model had a sensitivity of 80% and a specificity of 69%. This experiment demonstrated the potential of sweat, an unconventional sample, for lung cancer diagnosis 114.

### 4.7 Tissue

Compared with other methods, obtaining tissue samples is undoubtedly a more invasive method. However, tissue specimens also have unique advantages. Due to the differences in diet, living habits, and other aspects of various individuals, blood, urine, and other specimens are affected by many non-experimental factors. Tissue specimens can be collected from the same cancer tissue and adjacent tissue (or normal tissue), significantly reducing the influence of interference factors 21, 115.

In a study published in 2017, Chen et al. collected cancer tissue samples and para-cancer normal tissue samples from 32 cancer patients and analyzed the metabolomic signatures of cancer tissues using nuclear magnetic resonance spectroscopy. Multivariate statistical analysis showed that lipid, aspartate and choline metabolites in tumor tissues changed significantly at different time points. The authors believe that this technique will be useful in the diagnosis and staging of cancer 116.

According to a paper published in 2022, You et al. collected cancer and paracancerous tissues from 131 lung cancer patients and performed non-targeted metabolomics analysis based on LC-MS. Statistically, fatty acids, amino acids and most lysophospholipids were significantly increased in lung cancer tissues. However, 6-phosphogluconate, 3-phosphoglycerate, phosphoenolpyruvate, and citrate levels were decreased in lung cancer tissues. In addition, pathway enrichment analysis of lung cancer patients showed that energy, amino acid, and lipid metabolism were significantly disturbed 39. That same year, Mo et al. compared lung adenocarcinoma, benign pulmonary nodules and healthy tissue samples, using LC-MS to detect the three groups of samples and analyze their differential metabolites. The concentrations of metabolites such as creatine, glycerol and adenosine 3' -monophosphate were significantly different among the three groups. Further pathway analysis revealed that metabolites involved in central carbon metabolism pathway were most significantly altered in lung adenocarcinoma tissues, while disorders of protein digestion pathway were the main altered features of benign lung tumors 21.

### 4.8 Cell

In contrast to other samples taken from humans, the cells were grown in a controlled environment, meaning that non-experimental factors such as sex, age, lifestyle habits, and living environment were avoided. Therefore, by studying the metabolite differences between lung cancer cells and normal cells, the differential metabolites can be more accurately determined and the mechanism can be further investigated.

In the scholarly literature published by Filipiak et al. in 2019, the authors investigated the metabolites extracted from adherent cells of a lung cancer cell line. By utilizing CE-MS technology, the researchers were able to analyze metabolic processes such as the pentose phosphate pathway (PPP) and glycolytic metabolic pathway in lung cancer cell lines. Through this analysis, the study revealed that some intermediate metabolites within these metabolic pathways were significantly higher in concentration, while the concentration of some tricarboxylic acid (TCA) cycle intermediate metabolites was reduced. This method of analyzing metabolic pathways in cancer cell lines provides valuable insights into the pathogenesis of cancer and can lead to further discoveries in understanding the disease 117.

### 4.9 Summary on sample types

In general, the different sample types mentioned above had distinct characteristics. Collecting urine samples and VOCs in exhaled breath has significant advantages from a less invasive and painless perspective. Because the concentration of urine varies greatly throughout the day, a fixed time for urine collection should be imposed, and the same amount of water should be consumed. Urine samples should be collected before drinking water in the morning. Preservation of breath samples is difficult; therefore, testing should be performed immediately after sample collection. Tissue samples are more preferred from the perspective of reducing interference factors. Metabolomics studies using tissue samples usually do not require additional volunteers to act as control groups because tissues from lung cancer patients can be directly compared with those from healthy individuals. However, it is often difficult to access the patient's tissue. The collection of BAL and exhaled VOCs may be beneficial to finding more specific markers for lung cancer. Both sample types taken directly from the lungs and respiratory tract may detect metabolic markers more relevant to lung disease compared with blood, a biological fluid that permeates multiple systems in the body. In addition, unconventional sample types such as saliva and skin sweat have unique characteristics, which are convenient to collect and easy to be accepted by patients. The study of these samples also contributes to the early diagnosis of lung cancer (Table 1).

To integrate the advantages of various sample types, some scholars have collected multiple samples for metabolomic analysis. Ahmed et al. developed a new non-invasive detection method for lung cancer to search for novel biomarkers. Blood, urine and respiratory condensate were collected from lung cancer patients. The metabolite changes in lung cancer patients before and after surgery were detected and analyzed using NMR and mass spectrometry. The authors believe that the changes of metabolic substances in patients with lung cancer before and after surgical resection have potential clinical application value in lung cancer screening 118. Xu et al. considered polyamines as one of the most important biomarkers in cancer research. Plasma and urine samples were collected from patients with lung cancer and liver cancer, and these biological fluids were analyzed by UHPLC-MS. The concentration was then transformed into an independent variable by binary logistic regression analysis to determine the characteristics of patients. Significant independent variables were considered potential biomarkers and further verified by cluster analysis. Results showed that the ratio of putrescine to spermidine in plasma samples and the ratio of s-adenosine-l-methionine to n-acetylspermidine in urine samples could be successfully applied for the identification of patients with lung cancer and liver cancer 44. From this point of view, collecting multiple sample types in one study can help identify more sensitive and specific biomarkers to more reliably diagnose lung cancer and differentiate it from other diseases.

## 5. Summary and prospects

**Figure 1:**
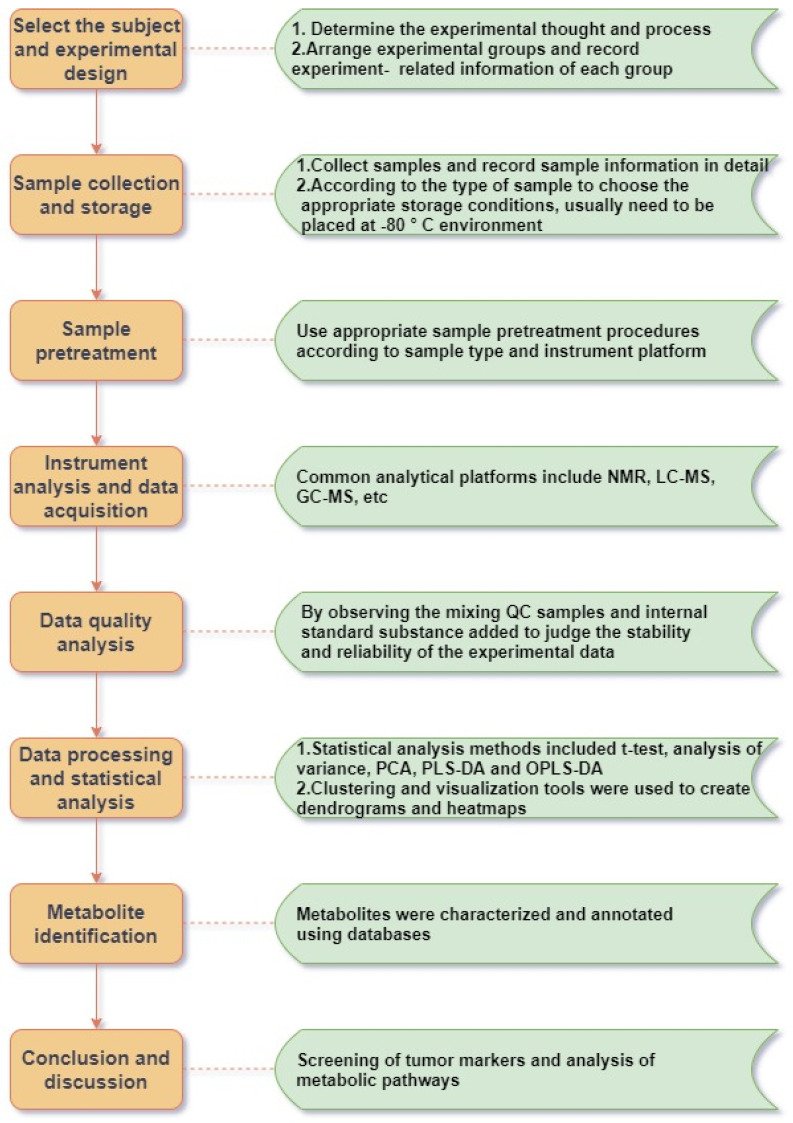
Schematic diagram of the metabolomics workflow.

Metabolomics is concerned with the increase and decrease of small-molecule metabolites in various matrix samples, which is the final result of the joint action of human genes and the environment, and contains abundant physiological and pathological information. Such studies usually include several steps such as sample collection and processing, instrumental analysis and detection, data processing and metabolic analysis (Figure 1). Metabolomics techniques can contribute to the search and development of biomarkers for various tumor diseases, including lung cancer. These biomarkers are promising tools for disease screening 40, 119, diagnosis 120, 121, exposure factors 122, 123, treatment 124, 125, prognosis 126 and mechanistic studies (Table 3).

In future studies, if conditions permit, the sample size can be expanded and non-experimental factors (such as age, sex, underlying diseases, diet, and other living habits) can be controlled to reduce the influence of these interfering factors on the experimental results. The accurate identification and characterization of metabolites from massive data in the untargeted metabolomics stage have always been difficult 127. The improvement of various metabolomics databases and the development of machine learning schemes, as well as the continuous improvement of the performance of various detection instruments, may provide more reliable means to accurately and efficiently find differential metabolites. Following untargeted metabolomics analysis, the addition of targeted metabolomics studies can enable further validation of the selected differential metabolites 128. Of course, differential metabolites are not equal to disease biomarkers, and rigorous medical validation is needed prior to their application in clinical practice.

In the latest research, with the continuous intersection of genomics, transcriptomics, proteomics, and metabolomics, multi-omics joint analysis has become a new research hotspot 69, 129. In addition, the emergence of spatial metabolomics has allowed researchers to more accurately understand the spatial location of metabolic changes in diseases 130, 131. Researchers can conduct joint analyses by integrating spatial metabolomics and multi-omics data to obtain more comprehensive and abundant biological information, and then conduct more in-depth mechanistic research on the disease.

## Figures and Tables

**Table 1:** Comparison of instrument platform characteristics and collection methods of each sample type

Sample type	Features	Collection method	Analytical technique	Ref
Blood	●Minimally invasive ●Sample collection is relatively easy ●Contains comprehensive metabolic information	Serum: ① Take blood samples from patients before breakfast. ② Centrifuge the blood samples at 3,500 rpm for 10 minutes at 4°C to obtain serum. ③ Freeze the serum sample at -80°C.	^1^H-NMR	42
		Plasma (EDTA used as anticoagulants): ① In the morning, draw 8 ml of blood from volunteers after an overnight fast into evacuated tubes containing EDTA as anticoagulant. ② Immediately separate plasma by centrifuging the blood samples at 4,500 rpm for 10 minutes at 4°C. ③ Freeze the plasma at -80°C.	GC-MS	24
		Plasma (heparin used as anticoagulants): ① Collect venous blood from the forearm and place it in a 10-ml Strek tube or a 10-ml heparin vacuum tube. ② Invert the tube 3-4 times, and centrifuge it at 450 g for 10 minutes at room temperature. ③ Drain the supernatant, and transfer the sample to a 0.5-ml storage tube. Store the tube at -80°C.	LC-MS	92
Urine	●Noninvasive, pain free ●Sample collection is convenient and easy	① Collect fasting morning urine from patients. ② Centrifuge urine samples at approximately 3,500 rpm for 15 minutes at 4°C within 2 hours, and isolate the supernatant. ③ Centrifuge the supernatant at approximately 3,000 rpm for 8 minutes at 4°C. Then transfer 1-2 ml of the supernatant to a 2-ml cryotube.	LC- MS	98
		① Collect morning urine samples from patients after an overnight fast in a sterile cup. ② Obtain 1 ml aliquot of the sample, and store it at -80°C freezer. ③ Before testing, the sample is thawed and centrifuged at high speed.	^1^H-NMR	97
Saliva	●Noninvasive, pain free ●Sample collection is convenient and easy	① Participants were asked not to eat or drink for at least 1.5 hours prior to saliva collection. ② Participants rinsed their mouths with water and were instructed to spit saliva into 50-cc Falcon tubes kept in paper cups filled with crushed ice. ③ Store the saliva sample at -80°C until further analysis.	CE-MS	104
Volatile organic compounds (VOCs)	●Noninvasive, pain free ●Metabolic information directly reflecting lung disease	① Before sample collection, instruct volunteers not to eat or smoke for several hours and remain in the room for at least 10 minutes. ② In a consultation room, have volunteers exhale their respiratory tract air into a 1-L Analytic Barrier Bag. ③ Immediately after exhaling, have them blow their alveolar breath into the same bag.	GC-MS	107
Bronchoalveolar lavage fluid (BAL)	●Unique value in diagnosing respiratory diseases	① Collect BAL samples from the lungs and bronchial lavage.Place each sample in a 1 ml Eppendorf tube. ② Store the Eppendorf tubes at -80°C.	GC-MS	43
Skin (sweat)	●Unique unconventional sample types ●Simple collection and high patient compliance	① Use a sweat inducer to heat the skin for 5 minutes. ② Cover the skin with a sweat collector to collect sweat for 15 minutes. ③ The collected sweat is stored at -80 ° C.	LC-MS	114
Tissue	●Cancer tissue and normal tissue can be collected simultaneously from the same individual, thereby greatly reducing the influence of interfering factors	① After surgical resection, rapidly excise each cancer tissue sample. ② Immediately freeze the cancer tissue sample in liquid nitrogen. ③ Store the tissue sample at -80°C until further analysis.	^1^H-NMR	116

**Table 2:** Comparison of instrument platforms

Instrument platform	Advantages	Disadvantages
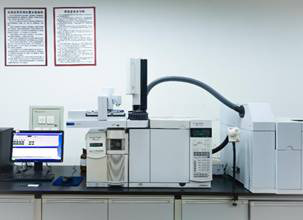 GC-MS	●Higher sensitivity and good separation ●Suitable for less polar substances ●Suitable for volatile substances	●Require tedious derivatization ●Not suitable for volatile samples
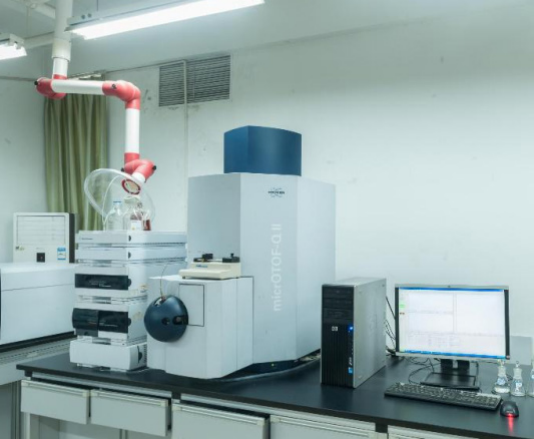 LC-MS	●Higher sensitivity and good separation ●Applicable to many types of samples ●No need for derivatization	●No standard metabolite database ●Chromatographic mobile phase must be configured in advance. ●Some chromatographic mobile phases may be harmful to humans or the environment.
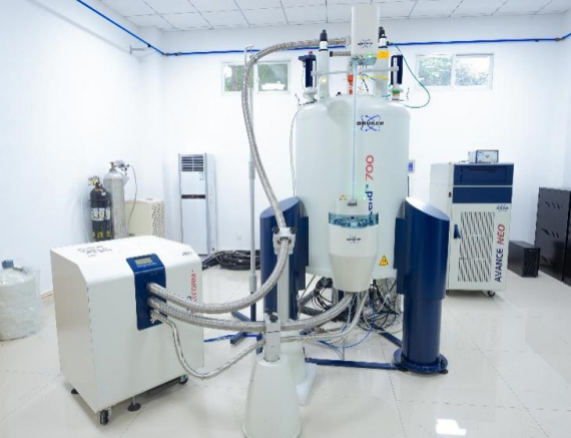 NMR	●Nondestructive testing without destroying the sample ●Simple sample preparation ●Can be used for liveness detection	●Lower sensitivity ●Limited dynamic range

**Table 3:** Metabolites in lung cancer

Year of publication	Sampletype	Grouping	Analytical technique	Main findings	Ref
2018	Serum	43HC,39 NSCLC	^1^H-NMR	(+)Proline, lactic acid, phenylalanine, alanine, tyrosine, glutamic acid, glycoprotein, and tryptophan (-) Glucose, glycine, glutamine, taurine, phosphocreatine, and threonine	42
2020	Serum	142 NSCLC, 159 HC	LC-MS	(+)C16-Sphinganine, Phytosphingosine, Sphinganine, Capric acid, and Arachidate (-) lysoPC(18:2), acyl-carnitine C10:1, and Inosine were decreased	69
2021	Serum	79NSCLC, 79HC	^1^H-NMR	(+) ADP, Diphospho-glycerate, Fructose-6-phosphate (-) ATP,NADP, 1,7-Dimethyl-xanthine	70
2023	Serum	193 NSCLC, 243HC	LC-MS	(+) 11,12-Epoxy-(5Z,8Z,11Z)-icosatrienoic acid, Cholic acid, 11- Deoxyprostaglandin, Glycocholic acid, and Docosahexaenoic acid ethyl ester (-) 2,4-Dihydroxybenzoic acid, Salicylic acid, and Piperine	71
2018	Plasma	43LC	LC-MS	Compared with the progressive disease group, the elevated metabolites in the partial response group were as follows: tryptophan, pyroglutamic acid, citric acid, succinic acid, phenylalanine, α-ketoglutaric acid, 2-hydroxyglutaric acid, and tyrosine	73
2021	Plasma	132PLC,47SLC, 77HC	^1^H-NMR	(+) Glucose, citrate, acetate, 3-hydroxybutyrate, and creatinine (-) Pyruvate, lactate, alanine, tyrosine, and tryptophan	74
2021	Plasma	57LC,59 HC	GC-MS	(+) 2-Hydroxybutyric acid (-) Cholesterol, oleic acid, inositol, and 4-hydroxybutyric acid	38
2016	Plasma and urine	50LC, 50 Liver cancer	LC-MS	The ratio of putrescine and spermidine concentration levels in plasma can be used to discriminate between liver and lung cancer.	44
2019	Serum and tissue	93LC,29HC	^1^H-NMR	(+) Lactate, glutamate, and glycerophosphocholine	35
2021	Urine and serum	29 urine samples and 32 serum samples (Sampling before and after surgery)	^1^H-NMR	(+)N6-methyladenosine (-)Leucyl proline and isopentenyladenine The variation range of urine metabolites before and after operation was greater than that of serum in patients with lung cancer	99
2014	Urine	469 NSCLC, 536 HC	LC-MS	(+)N-acetylneuraminic acid, creatinine riboside	96
2019	Urine	275 female LC, 278 female HC	^1^H-NMR	(-) 5-methyl-2-furoic acid Air pollution increases the risk of lung cancer	97
2020	Urine	68HC,72LC	LC- MS	(+) Glycyl-glycine, 4-pyridoxic acid, crithmumdiol, 1-methylhistidine, tryptophan, and glutamine (-) 8-Hydroxynevirapine and indoxyl sulfate	98
2020	DBS	37SCLC,37HC	LC-MS	(+) Ceramide and sphingomyelin (-) Glutamate, riboflavin, serotonin, hypoxanthine, cholic acid, arachidonic acid, ethanolamine, and L-aspartic acid	82
2017	VOCs	107LC,29HC	GC-MS	The combination of CHN, methanol, CH3CN, isoprene, and 1-propanol can diagnose lung cancer, but it does not indicate the increase or decrease in the level of a single substance.	107
2016	BAL	24LC,31HC	GC-MS	(+) Acetic acid, palmitic acid, and stearic acid (-) Lactic acid, glycerol, L-glycine, L-aspartate, L-proline, L-glutamine, benzoic acid, fructose, phosphoric acid, isocitric acid, inositol, and galactoseInosine, oleic acid, and cholesterol	43
2013	Tissue	9LC	CE-MS	(+) Lactic acid, phosphofructokinase, and pyruvate kinase. Most amino acids, especially branched-chain amino acids	19
2017	Tissue	32LC	^1^H-NMR	(+) Lipids, aspartate, and choline-containing	116
2020	Tissue	131LC	LC-MS	(+)Almost all amino acids and most lysophospholipids, fatty acids (-) 3-Phosphoglyceric acid, 6-phosphogluconic acid, phosphoenolpyruvate and citric acid	39
2020	Tissue	10 AdLC , 10HC	LC-MS	Differential metabolites such as glycerol, creatine and 3-monophosphate can be used for the diagnosis and prognosis of lung cancer	21
2019	Cell	Lung cancer adherent cell line	CE-MS	The concentration of some intermediate metabolites in pentose phosphate pathway (PPP) and glycolytic metabolic pathway in lung cancer cell lines was significantly higher, and the concentration of some intermediate metabolites in tricarboxylic acid (TCA) cycle was decreased	117
2015	Skin (sweat)	41LC, 55HC	LC-MS	nonanedioic acid, suberic acid, a trihexose, a tetrahexose and the monoglyceride MG(22:2) can be used to distinguish between healthy and lung cancer patients	114
2021	Saliva	89 early-stage LC, 11 advanced-stage LC, 50 HC	TELDI-MS	Screening out 23 metabolites mainly related to amino acid and nucleotide pathways	103
2022	Saliva	41 LC, 21 benign lung lesions	CE-MS	Diethanolamine, cyclosine, lysine, and tyrosine can be used to distinguish between lung cancer and benign lung lesions	104

Abbreviations in this table: (+) Elevated markers (compared with healthy controls), (-) reduced markers HC, healthy control; LC, lung cancer; PLC, primary lung cancer; SLC, secondary lung cancer; SCLC, small-cell lung cancer; NSCLC, non-small-cell lung cancer; SqLC, squamous cell carcinoma of the lung; AdLC, adenocarcinoma of the lung; LCCL, lung cancer cell line; DBS, dried blood spot; VOCs, volatile organic compounds; BAL, bronchial lavage fluid; NADP, nicotinamide adenine dinucleotide phosphate
